# The Role of the Glucocorticoid Receptor and Its Phosphorylation in Neurological Disorders

**DOI:** 10.3390/ijms26094213

**Published:** 2025-04-29

**Authors:** Saranya Gadwala, Chaitali Ghosh

**Affiliations:** 1 Neurovascular Research, Department of Biomedical Engineering, Lerner Research Institute, Cleveland Clinic, Cleveland, OH 44195, USA; gadwals@ccf.org; 2 Department of Biomedical Engineering and Molecular Medicine, Cleveland Clinic Lerner College of Medicine of Case Western Reserve University, Cleveland, OH 44195, USA

**Keywords:** glucocorticoid receptor, GR phosphorylation, glucocorticoid, brain disorders, blood–brain barrier, drug therapy

## Abstract

Hormone-dependent phosphorylation of steroid receptors is a mechanism for modulating glucocorticoid receptor (GR) transcriptional responses. Evidence indicates that GR phosphorylation can influence receptor transcriptional activation in a gene-specific manner, which could have positive or negative impacts, where the relative level of phosphorylation is an important determinant of overall GR function. This review provides insights into the regulatory mechanism of GR phosphorylation in the brain, cellular and molecular specificity affecting neurovascular function, and the impact of GR phosphorylation in neurological disorders. Furthermore, the role of various endogenous and exogenous factors and sex-dependent associations with GR functional changes due to phosphorylation and other interlinking mechanisms are considered. Finally, we highlight the potential therapeutic approaches which have been evaluated, while challenging GR phosphorylation and the overall influence on the activity of GR in brain disorders.

## 1. Introduction

Glucocorticoids are a class of steroid hormones, released in stress responses to aversive stimuli. They play a role in basal- and stress-homeostasis and are produced by the hypothalamic–pituitary–adrenal (HPA) axis. The HPA axis is activated in times of stress, contributing to various psychiatric disorders [1] and leading to structural and neurological changes. Glucocorticoids also play a role in regulating many different biological processes, as well as influencing physiological functions. They can be used therapeutically as immunosuppressive agents to manage a range of inflammatory, autoimmune, and lympho-proliferative diseases [2], and are regulated molecularly by a receptor protein known as the glucocorticoid receptor (GR). The GR can be phosphorylated at multiple residues, which can significantly affect crucial functions such as GR turnover, subcellular trafficking, target promoter specificity, cofactor interaction, and receptor signaling and stability [2]. GR phosphorylation is an intricate process, with many implications that are not yet fully understood. Pathophysiological conditions linked to GR trafficking and transcription may be sustained by changes in GR phosphorylation, which could be modulated by pharmacological intervention.

## 2. Regulatory Mechanism of GR Phosphorylation in Brain

The GR regulates the gene expression of glucocorticoid genes, which make up about 3–10% of the human genome [2], and the GR belongs to the family of steroid and thyroid-activated intracellular transcription factors [2]. The GR is ubiquitously present in almost all human tissues and organs, working as a ligand-dependent transcription factor which runs developmental and metabolic responses to glucocorticoids. Ligands are usually cytoplasmic before they are bound to the GR and a heat-shock protein 90-p23 chaperone complex. This complex inactivates the GR’s DNA binding and transcriptional regulation responses, while maintaining it as ready for high-affinity ligand binding [3,4,5]. The GR also contains three major functional domains, known as the N-terminal domain (NTD), DNA binding domain (DBD), and the ligand-binding domain (LBD). An amino acid ‘hinge’ region lies between the DBD and LBD, and between the NTD and LBD there are the two transcription factors, AF1 and AF2 [6]. The AF1 site is a crucial hub for GR phosphorylation, containing the serine residues which are main players in GR phosphorylation [6,7].

**Figure 1:**
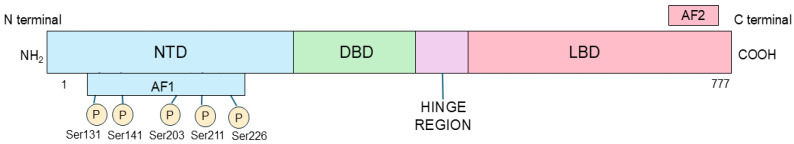
Schematic representation of the GR, outlining the different domains and significant serine phosphorylation residues.

One of the main mechanisms by which ‘a cell’ can signal is through the posttranslational addition of a phosphate group to various proteins, or the process of phosphorylation. Due to this reversible process, the presence or absence of a phosphate group on certain amino acid residues may offer information on how those proteins are acting, as well as about their downstream GR signaling. As a transcription factor, when a hormone is bound to GR (Figure 1), it can be phosphorylated at several sites which are known to be highly conserved in both humans and rodents.

GR phosphorylation is critical to receptor activation because it influences important functions of the GR, including ligand binding, subcellular localization, half-life, transcriptional activity, and DNA binding [8,9]. The GR becomes phosphorylated (Figure 1) at multiple serine/threonine residues [10]. The GR’s N-terminal domain includes the phosphorylation sites at the amino acid serine, including Ser131, Ser141, Ser203, Ser211, and Ser226 [11]. Within the GR’s AF1 domain, the GR is phosphorylated at the crucial serine sites of Ser203, Ser211, and Ser226. The interplay between phosphorylation of different serine residues shows relationships that can be inverse, suggesting that the GR can be phosphorylated simultaneously at multiple residues, often with the activity of one affecting the other [11].

### 2.1. Ser203

Findings on Ser203 suggest that the portion of the GR phosphorylated at the Ser203 site is only contained in the cytoplasm. This suggests that the GR phosphorylated at Ser203 may not be transcriptionally active, and additional evidence has shown that the GR phosphorylated there is not localized in the nucleus [11]. A mutation to the Ser203 site decreasing the phosphorylation at Ser211 indicates a possible interdependence between these two sites, and Ser203 may need to be phosphorylated first before phosphorylation at Ser211 can efficiently occur [12]. There may also be an inverse relationship between Ser203 and Ser226, with each site gatekeeping the activity of the other [13]. It has been shown that Ser203 is phosphorylated by cyclin E/cyclin-dependent kinase 2 and cyclin A/Cdk2, and dephosphorylation at this site is regulated by protein phosphatase 5 [3].

### 2.2. Ser211

Ser211 could potentially serve as a biomarker for the active GR in vivo, as through experimentation with various agonists and antagonists, a correlation has been observed between the amount of phosphorylation at Ser211 and the transcriptional activity of the GR [12]. Phosphorylation at Ser211 promotes GR translocation to the nucleus and enhances transcriptional activity, while phosphorylation at Ser226 inhibits transcriptional activity. This makes the ratio of pGR-Ser211/pGR-Ser226 a critical parameter in determining the GR-dependent transcriptional activity and expression of GR-responsive genes [12]. Specific residues on the GR can be phosphorylated by certain kinases, and p38 in the Mitogen-activated protein kinase (MAPK) pathways has been shown to be an effective kinase for GR phosphorylation at Ser211 [6]. Ser211 has been suggested to have an inverse relationship with Ser203, and while Ser203-phosphorylated GR may be localized to the cytoplasm, Ser211-phosphorylated GR was found in the nucleus, suggesting localization there [12].

### 2.3. Ser226

Ser226 may function in an inverse relationship with both Ser203 and Ser211. Blocking Ser226 phosphorylation has been shown to increase GR’s transcriptional activity, and GR-phosphorylated Ser226 (pGR-Ser226) has been associated with the endogenous GR-containing promoters tyrosine aminotransferase and sulfonyl transferase 1A1 [11]. The GR exhibits its greatest transcriptional activity when the relative phosphorylation at Ser211 exceeds that of Ser226, and replacing the Ser226 residue with an alanine has been shown to increase the GR’s transcriptional response [3]. GRα phosphorylated at Ser226 spends less time in the nucleus, due to either greater cytoplasmic retention or enhanced nucleocytoplasmic transport [14]. Phosphorylation of Ser226 by c-Jun N-terminal kinase (JNK) inhibits GR transcriptional activity and regulates GR export from the nucleus during hormone withdrawal [3].

GR phosphorylation is highly specific to the type of cell, tissue, species, or promoter. This can likely be explained by the sheer variety of serine/threonine kinases and phosphatases that can partake in GR phosphorylation [9]. The GR’s phosphorylation status (Table 1) can affect GR-dependent transcriptional activity, GR stability, and nucleo-cytoplasmic shuttling, which can either enhance or inhibit GR-induced gene expression and influence overall glucocorticoid sensitivity [11].

## 3. Impact of GR Phosphorylation at the Cellular and Molecular Level Affecting the Neurovascular Interface

The neurovascular interface between the tissue of the central nervous system and circulating blood plays a vital role in maintaining a healthy brain by facilitating transport of ions, small molecules, and cellular regulators in and out of the brain tissue [16]. The blood–brain barrier (BBB) is made of endothelial cells that line up along the cerebral microvasculature, and it helps keep the brain in a stable state, protecting it from fluctuations imposed by changes in plasma composition, and circulating neurotransmitters and xenobiotics that can disturb neural function. By maintaining a stable state of homeostasis in the brain, the BBB helps regulate the controlled activity of neurons [17,18].

Glucocorticoid activity has been shown to influence BBB structure and integrity, via upregulating tight-junction proteins in the endothelial cells of brain vasculature, and GR activity may suggest a reduction in the expression of the vascular endothelial growth factor (VEGF). The VEGF can induce vascular remodeling in the brain, suppress endothelial cell proliferation, and enhance pericyte coverage of brain microvessels. Thus, using the GR to promote vasodilation involves VEGF pathways and can cause brain tissue edema [5].

Glucocorticoids have been shown to play a key role in clinically managing various CNS disorders associated with a compromised BBB, including edema, brain tumors, and multiple sclerosis [19]. When corticosteroid hormones bind to the GR, it activates the GR as a transcription factor, and this changes the organism’s neurovasculature [20]. For example, the corticosteroid dexamethasone strengthens the BBB by initiating GR-mediated signaling, which upregulates tight junction proteins such as occludin, claudin-5, or ZO-1, and increases transendothelial electrical resistance [4,19,21]. Dexamethasone treatment has also been shown to increase barrier integrity via increasing angiopoetin-1 and decreasing the vascular endothelial growth factor [22]; however, these protective effects may be disturbed during hypoxic conditions, such as when the GR undergoes proteasome-dependent degradation [23].

Regarding the specific effects of GR phosphorylation on the BBB, this is a relatively underdeveloped area of research and warrants further investigation. Existing research has shown that GR activation, not necessarily through phosphorylation, may enhance BBB integrity by upregulating tight junction proteins like occludin and claudin-5, which are key to upholding BBB structure and limiting permeability [24]. It was noted in in vitro studies that treatment with corticosterone and dexamethasone, as a GR agonist, reduced GR expression in astrocytes, whereas RU486, a GR antagonist, inhibited both astrocyte proliferation and GR expression [25]. In the follow-up study, the same group found that the effect of excessive glucocorticoid release affected GR expression and the number of astrocytes in vivo, by administering adrenocorticotropic hormone to rats for 14 days. GR expression was found to be reduced in the prefrontal cortex and hippocampus, and the number of astrocytes was reduced in the frontal cortex. Overall, the results suggested that glucocorticoids decrease the number of astrocytes by reducing GR expression. Glucocorticoids have also been shown to suppress inflammatory signals and strengthen the functioning of the endothelial barrier function, restoring endothelial–astrocyte connections, which could together protect the neurovascular damage under disease state [19,23,26].

Although these findings establish that the GR is a crucial regulator of the BBB’s integrity, the role of GR phosphorylation in these processes is still poorly understood. It is well known that GR phosphorylation is a post-translational modification which influences GR function (Table 2), affecting nuclear translocation, transcriptional activity, and interaction with coregulators [14]. As GR phosphorylation has been shown to play a role in neurodegenerative diseases and cognitive dysfunction, it is possible that it could influence the dynamics of the BBB, but there is sparse direct evidence of how it does so, and the mechanistic underpinning is understudied.

## 4. GR Phosphorylation in Brain Disorder

### 4.1. Epilepsy

Epilepsy is a chronic neurological disorder characterized by frequent, unprovoked seizures. Patients with epilepsy often also suffer from memory impairment, and in temporal lobe epilepsy (TLE), the most common drug-resistant epilepsy, alterations to the mesial temporal lobe’s structure may directly correlate to memory disturbance [28], as summarized in Table 3. Glucocorticoids and stress can affect memory in different ways, with acute stress leading to lucid memory of certain events, but chronic stress correlating with impaired memory, as well as leading to the development of neurodegenerative diseases.

Synaptic plasticity is a process crucial to memory formation, and an important feature of this is long-term potentiation (LTP). During LTP, the repeated firing of neurons increases certain synaptic connections regulated by the GR [28]. In animal models, it was found that when temporal cortex specimens were treated with dexamethasone, LTP was impaired in epileptic patients. In a recent study, we found that the GR, heat shock protein 70 (Hsp70), heat shock protein 90 (Hsp90), and heat shock protein 40 (Hsp40) were upregulated in epileptic brain regions compared to non-epileptic brain regions. More specifically, increased GR and Hsp90 co-localization were observed in microvessels, astrocytes, and neurons of epileptic tissue, while GR and Hsp70 co-localization were observed in the microvessels and neurons [4]. Decreased levels of Hsp70 and Hsp90 were bound to the GR in human epileptic (dysplastic) brain tissues compared to nonepileptic (non-dysplastic) tissues. A similar pattern was found in human epileptic brain endothelial cells versus non-epileptic brain endothelial cells. As unbound GR-Hsp is indicative of complete GR maturation, decreased GR-Hsp interaction in epileptic tissue reflects accelerated GR maturation. The corresponding increase in ATPase activity in epileptic tissue suggests increased chaperone activity, demonstrating the essential role of heat shock proteins in the acceleration of GR maturation. However, the role of GR phosphorylation specifically in the epileptic brain and its function remains unclear.

In some cases of experimental and clinical epilepsy, it is not possible for seizures to be controlled by antiepileptic drugs. Inflammation may be present, but it is multifactorial and can be caused by various factors. This increases the complications in finding a remedy which neutralizes the right proinflammatory factors to reduce seizure activity [29]. Glucocorticoids have therapeutic potential, as they are known to activate a broad anti-inflammatory response, and specifically the annexin A1 (ANXA1)–GR axis, which is an endogenous anti-inflammatory effector. ANXA1 is produced during GR phosphorylation and nuclear translocation by peripheral, CNS immune, and endothelial cells. In 2019, Zub et al. administered ANXA1 exogenously in an in vivo model of temporal lobe epilepsy and found a decrease in phosphorylated GR (pGR) and pGR/total-GR protein expression in the hippocampus during epileptogenesis, and a continued decrease up to seizure progression and spontaneous recurrent seizure [29]. This reduction in pGR and decrease in the functionality of the GR–ANXA1 pathway during experimental seizure progression poses the possibility of further investigating GR desensitization in epileptic subjects who fail to respond fully to GR treatments.

### 4.2. Stroke

Stroke is a condition where blood flow to the brain is interrupted, resulting in brain cell death. Both preclinical and clinical studies of stroke have shown disruption in the regulation of the HPA axis. The HPA axis’ engagement, along with glucocorticoid signaling, influences the severity of neuronal damage and functional impairment, as well as the recovery trajectory for stroke patients. Stress and high glucocorticoid levels are associated with increased morbidity and a poor prognosis for stroke patients [30,31]. A reason for poor stroke outcomes is blockade of the GR, preventing post-ischemic lymphocytopenia [32]. Stress and high glucocorticoid levels are also associated with poor stroke outcome and morbidity, making GR regulation a potential avenue for stroke treatment [33]. Additionally, systemic inflammation is another poor outcome of stroke, and glucocorticoids play a key role in inhibiting inflammation [34]. If glucocorticoids fail to inhibit inflammation, studies suggest that this may result in further disease development, and the biological response to glucocorticoids is dependent on factors like the glucocorticoid concentration and individual differences in glucocorticoid sensitivity [34].

Clinical and rodent model data have demonstrated that glucocorticoids of the HPA axis are involved in ischemic-stroke-induced brain dysfunction [35]. Ischemic stroke caused by acute brain transient ischemic attack creates a complicated sequence of events where the nervous system and HPA axis may ultimately result in brain damage [35]. It has been shown in animal studies that increased activity of glucocorticoid-inducible kinase 1 aggravates ischemic brain damage [34]. In a rat model following ischemic stroke receiving daily restraint stress for four weeks, stress and stroke enhanced GR activation in the ischemic lesion, as well as diminished limb use and hindered motor recovery [33]. Though glucocorticoids have been known to have stabilizing and ameliorating effects on the BBB and tissue edema in certain neoplastic and inflammatory disorders in the CNS, they mostly seem ineffective in the presence of ischemic stroke [23].

Inhibition of the proteasome may offer an avenue to overcome glucocorticoid resistance in a hypoxic BBB, as it was found in a murine model that while dexamethasone treatment failed to reverse conditions of hypoxia, inhibition of the proteasome fully restored the BBB’s original properties during O_2_/glucose deprivation [23]. High-dose corticosteroids have been shown to acutely increase the activity of endothelial nitric oxide synthase (eNOS), which contributes to the production of vascular nitric oxide (NO) [20]. Vascular NO production has been shown to increase vascular integrity, regulating cerebrovascular perfusion, and protect against stroke by increasing collateral flow to the ischemic area, and through the PI3K/Akt pathway, non-nuclear GR rapidly activates eNOS and contributes to protecting against stroke [20].

### 4.3. Brain Tumor

Brain tumors are caused by abnormal cell growth around the brain and can be implicated in diseases like brain cancer. Glucocorticoids have long been used in treating brain tumors, and they are one of the most powerful agents in reducing tumor-related edema, but they are also associated with adverse side effects [36], as seen in Table 3. Through GR signaling depending on the time of day and the clock genes *Bmal1* and *Cry*, daily glucocorticoids have been shown to alter the growth of glioblastoma (GBM), one of the most common malignant brain tumors in adults and with a poor prognosis [37]. This may be due to the nature of glucocorticoids having one of the highest amplitude circadian outputs and being able to synchronize circadian clocks in tissues like the brain, liver, kidney, and heart, since GBM tumors may act as peripheral circadian “pacemakers” and synchronize their daily rhythms with those of the host in order to regulate tumorigenic processes [37]. Human GBM samples in the study showed that high GR expression acutely increased mortality risk [37]. Dexamethasone, through GR activity, has an inhibitory effect on cancer stem cell proliferation, and can also inhibit astroglial differentiation from neural precursor cells [36].

### 4.4. Traumatic Brain Injury (TBI)

Traumatic Brain Injury (TBI) refers to brain dysfunction caused by an external force, usually a large, violent blow to the head. Survivors of TBI can experience persisting psychiatric symptoms. TBI induces stress responses by activating the HPA axis, and restraint stress has been used in psychological models to reflect this. In a mouse model, following 1 h of acute restraint stress, the mouse showed increased GR phosphorylation, which could influence downstream GR activity. A recent review summarized clinical and experimental studies that explored the use of synthetic glucocorticoids as a therapeutic for stress-related TBI outcomes, but these yielded mixed results. Furthermore, GR-mediated stress dysfunction after TBI, highlighting the role of microglia, and studies which target microglial GR in the context of stress and injury and cell-specific GR interventions may be a promising strategy for long-term TBI pathophysiology [38]. In another report on TBI association with psychiatric dysfunction and increased alcohol use, alcohol-drinking, post-TBI rats treated with the drug JZL184, known to attenuate increased neuroinflammation and neuronal hyperexcitability at the site of injury as well as improve neurobehavioral and neurological severity score, were shown to have increased levels of phosphorylated GR in the central nucleus of the amygdala [39]. Collectively, these studies indicate that mild or chronic TBI is impacted by GR phosphorylation, regulating brain function.

### 4.5. Neurodegenerative Disorders

#### 4.5.1. Alzheimer’s Disease

Alzheimer’s Disease (AD) is the most common form of dementia, characterized by memory loss, decreased cognitive function, and the aggregation of amyloid-beta proteins in the brain. Abnormal cortisol levels and GR activation play a role in age-related progression of AD [40]. Glucocorticoid resistance poses a risk factor for AD, and GR phosphorylation through brain-derived neurotrophic factor (BDNF) signaling is involved in remodeling synaptic structure and plasticity [41]. BDNF is associated with a group of neurotrophic growth factors related to controlling neogenesis, neuronal survival, and long-term potentiation, and is influenced by GR modulation [5]. Deletion of GR phosphorylation at BDNF-responding sites and downstream signaling through the MAPK-phosphatase DUSP1 triggered tau phosphorylation and dendritic spine atrophy in a mouse model [41]. Of the genes depending on BDNF and GR phosphorylation, DUSP1 expression is associated with suppressing hyperphosphorylated tau and synaptic loss in the mouse cortex, making it a possible target for therapeutic intervention [41].

Disrupting GR phosphorylation at the BDNF-dependent sites Ser134 and Ser267 in an APP/PS1 mouse model with an early-phase AD-like progression aggravated the deleterious effects of the APP/PS1 genotype on mortality, neuroplasticity, and cognition, without affecting amyloid-β deposition or vascular pathology [40]. The loss of the neutrophin-mediated pathway aggravated the harmful effects of the brain cortisol response, which the authors suggested may contribute to the onset or progression of AD, making drugs that specifically increase GR phosphorylation at neurotrophic sites an important target for therapeutic intervention [40]. As also shown in the article by Arango-Lievano et al. [42], BDNF, in the presence of glucocorticoids, phosphorylates the GR at sites that facilitate its translocation to the cell nucleus for transcriptional actions, and this effect is synergistic with the ability of glucocorticoids to activate, via a genomic mechanism, the phosphorylation of the TrkB receptor independently of BDNF, thus creating a positive feedback loop.

#### 4.5.2. Huntington’s Disease

Huntington’s Disease (HD) is a fatal genetic neurodegenerative disorder characterized by a CAG triplet repeat expansion in the human huntingtin (HTT) gene, which leads to an increase in function of the mutant huntingtin protein (mHTT) [43]. HD patients have shown significant elevation in levels of circulating glucocorticoid cortisol, as glucocorticoids have been shown to modulate mHTT inclusion formation [43]. This could make GR antagonism a potential pathway for treating HD [44]. In a R6/2 HD mouse model, normalization of glucocorticoids significantly improved neuropathological readouts and led to partial prevention of global brain atrophy, altered neurogenesis, and a decrease in mutant huntingtin inclusion burden [43]. The selective GR antagonist CORT113176 was shown to delay several motor and neuropathological symptoms of HD in a male R6/2 mouse model [44]. Huntingtin-associated protein 1 (Hap1) binds to huntingtin and interacts with GRs in mouse hypothalamic neurons [45]. Hap1 has been shown to stabilize the GR in the cytoplasm, and Hap1 dysfunction may alter stress response in animals [45].

#### 4.5.3. Multiple Sclerosis

Multiple Sclerosis (MS) is an inflammatory disease which impairs CNS. Among groups of MS patients, the negative feedback system run by glucocorticoids and the HPA axis is dysregulated, with increased basal secretion of cortisol and an increased size of adrenal glands [46,47]. Due to the anti-inflammatory and immunosuppressive properties of glucocorticoids, they are usually recommended as first-line therapy for acute MS attacks [48]. Exogenous glucocorticoids are commonly used to treat MS relapses, but responses vary among patients, which is likely due to differences in sensitivity to glucocorticoids [46,47,49]. GRα, a splice variant of GR, along with the chaperone FK506-binding protein (FKBP5) influence glucocorticoid cellular sensitivity and, in Chinese MS patients, it was found that GC-sensitivity was linked to higher levels of GRα and lower levels of FKBP5 [48]. Glucocorticoids have the capacity to suppress the pathogenic immune response in MS by polarizing monocytes towards an anti-inflammatory phenotype and amplifying their migration to the inflamed CNS [50].

### 4.6. Psychiatric Disorders

By modulating brain structure and function, glucocorticoids can influence perception, cognition, and mood [41]. Stress-mediated peaks caused by glucocorticoids are a major feature in depression, and although patients with depression have high glucocorticoid levels, their GR signaling is dysfunctional [41]. GR phosphorylation is shown to be BDNF-sensitive at the sites S155, S287, and S246 through the TrkB-MAPK pathway, and disruptions to this process could play a role in stress-related disorders [41]. Chronic stress has been shown to reduce BDNF levels, which reduces GR phosphorylation and plasticity, and enhancing GR phosphorylation could represent a potential therapeutic approach for psychiatric disorders like depression [41].

#### 4.6.1. Metabolic Syndrome (MetS)

Metabolic syndrome, consisting of dyslipidemia, dysglycemia, visceral obesity, and hypertension, frequently accompanies psychiatric disorders, as it has been shown that depression and anxiety is more prevalent in individuals with MetS than the general population [51]. Decreased BDNF/TrkB agonist-dependent GR phosphorylation may contribute to comorbidity of MetS along with psychiatric disorders like anxiety and depression, making the BDNF/TrkB pathway a target for potential therapeutic recovery [51].

#### 4.6.2. Major Depressive Disorder (MDD)

Impaired GR phosphorylation may also contribute to the pathophysiology of depression, specifically major depressive disorder (MDD) [12]. GR phosphorylation at the Ser211 site enhances transcriptional activity, while GR phosphorylation at Ser226 inhibits transcriptional activity. MDD patients displayed an increase in GR phosphorylation at Ser226, and a smaller increase in phosphorylation at Ser211 compared to controls, and the pGRSer211/pGRSer226 was lower in MDD patients, implying reduced levels of transcription [12].

#### 4.6.3. Bipolar Disorder (BD)

Abnormal GR functioning is observed as a key factor of bipolar disorder (BD), and BD patients have shown decreased glucocorticoid responsiveness, which could be due to reduced GR number/function in lymphocytes and brain tissue, as well as a lessened ability of the GR to bind to DNA in lymphocytes [11]. BD patients have reduced total GR phosphorylation, but phosphorylation at the Ser211 site is significantly increased, especially in depressive states, suggesting that GR phosphorylation may play a role in BD pathophysiology [11].

**Table 3:** GR phosphorylation’s (pGR) role in brain disorders and potential therapeutic targets.

Brain Disorder	Changes in GR Phosphorylation	Effects on Pathology	Potential Therapeutic Targets	References
Epilepsy	↓ pGR-Ser203, ↓ pGR-Ser211	Impaired anti-inflammatory response, GR desensitization	Enhancing pGR, ANXA1-GR axis	[29]
Ischemic Stroke	Dysregulated pGR, GR degradation in hypoxia	Increased inflammation, BBB disruption, impaired recovery	Proteasome inhibitors may restore GR function	[23]
Brain Tumors (Glioblastoma)	Circadian linked pGR influences tumor progression	High GR expression worsens prognosis, dexamethasone reduces peritumoral edema	Glucocorticoid therapy timed in sync with circadian activity	[36,37]
Alzheimer’s Disease (AD)	↓ pGR at neurotrophic sites Ser134, Ser267	↑ Tau phosphorylation, synaptic loss, cognitive decline	BDNF signaling to enhance neuroprotective GR phosphorylation	[41]
Huntington’s Disease (HD)	↑ Cortisol, altered pGR	↑ Mutant huntingtin toxicity, brain atrophy	GR antagonists (CORT113176) to reduce neurotoxicity	[44]
Multiple Sclerosis (MS)	Dysregulated pGR and sensitivity	Impaired glucocorticoid response, neuroinflammation	Enhancing GR response via GRα and FKBP5 modulation	[46,47,48]
Major Depressive Disorder (MDD)	↑ pGR-Ser226, ↓ pGR-Ser211/pGR-Ser226 ratio	Reduced GR-induced transcriptional activity, HPA axis dysfunction	Enhancing pGR at Ser211, FKBP5 modulation via antidepressants	[12]
Bipolar Disorder (BD)	↓ Overall, pGR, ↑ pGR-Ser211 in depressive states	Altered stress response, glucocorticoid resistance	GR modulation for phase-dependent treatment	[11]

[Upregulation indicated by ↑ and downregulation indicated by ↓]. Abbreviations: pGR, GR phosphorylation; Ser, serine; ANXA1, Annexin A1; BBB, blood–brain barrier; BDNF, brain-derived neurotrophic factor; FKBP5, FK506 binding protein 51 also called FKBP5; HPA axis, hypothalamic–pituitary–adrenal axis.

## 5. Factors Influencing GR Phosphorylation

### 5.1. Exogenous Factors

#### 5.1.1. Protein Phosphatase Inhibitors

Treating cells with okadaic acid, a protein phosphatase inhibitor targeting protein phosphatase 2A and protein phosphatase 1, leads to the hyperphosphorylation of the GR, as well as the accumulation of the GR in the cytoplasm [52]. Calyculin A, another protein phosphatase inhibitor, is more potent than okadaic acid, and geldanamycin, which inhibits the heat shock protein 90 complex, also increases basal GR phosphorylation [52]. When phosphatase inhibitors like okadaic acid and calyculin A influence GR activity, they increase GR phosphorylation [53].

#### 5.1.2. External Signaling Molecules

The GR can also be influenced by exogenous factors such as serum, growth factors, and constitutively active signaling proteins such as racQ61L through activation of the c-Jun N-terminal kinase (JNK) and extracellular-signal-regulated kinase (ERK) pathways [54].

#### 5.1.3. MAPK Pathway Activation by External Stimuli

The three pathways in the MAPK family of p38, JNK, and ERK all differentially modulate GR activity and phosphorylation [53]. JNK and ERK activation inhibits GR transcription enhancement, and using inhibitors to inhibit JNK and ERK enhances GR phosphorylation [53]. The ERK and JNK pathways are both thought to phosphorylate the nonpolar-X-Ser/Thr-Pro sequence motif, but the ERK pathway is activated by growth factors through Ras activation, and JNK is activated by UV radiation, tumor necrosis factor α, proinflammatory cytokines, and the lipopolysaccharide of Gram-negative bacteria [54]. ERK inhibits the enhancement of GR transcription, while inhibiting JNK and ERK with inhibitors can enhance GR function [53].

#### 5.1.4. UV Irradiation

UV irradiation treatment, which is used to activate the MAPK pathway, influenced GR phosphorylation at sites Ser226 and Ser211, generally decreasing the quantity of phosphorylated GR isoforms [55].

### 5.2. Endogenous Factors

#### 5.2.1. Heat Shock Protein 90 Complex

A GR-derivative without the ligand-binding domain can no longer bind the heat shock protein 90 complex, which increases GR phosphorylation at the three major sites Ser203, Ser211, and Ser226 [52].

#### 5.2.2. Kinases (CDKs, JNKs)

**Figure 2:**
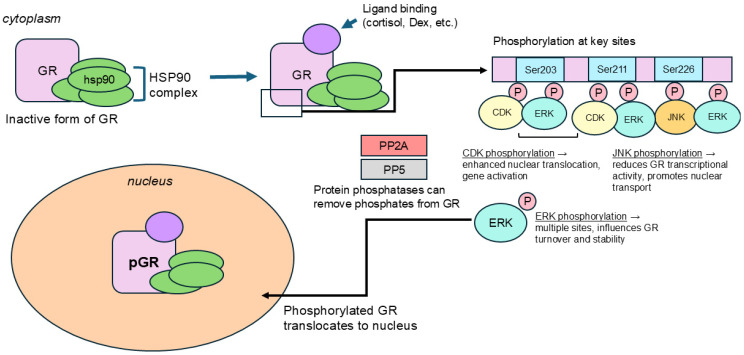
Schematic representation of how different kinases (CDK, ERK, JNK) and phosphatases modulate GR phosphorylation.

Cyclin-dependent kinases (CDKs) phosphorylate GR, modulating its activity. Cyclin-dependent kinases target GR phosphorylation at the site Ser211 to enhance transcriptional activity, which can be opposed by the JNK pathway phosphorylating the GR at Ser226, which reduces transcriptional activity, and the balance between cyclin-dependent kinases, and JNK helps regulate GR’s apoptosis and gene expression [55], as depicted in Figure 2. In rats, CDK2 phosphorylates rat GR at Ser224 and Ser232, while CDK5 suppresses the transcriptional activity of GR by reducing the binding of transcriptional cofactors to GR-responsive promoters [53]. The GR may cycle through a phosphorylation/dephosphorylation cycle which maintains steady-state receptor phosphorylation at a low basal level even in the absence of a ligand, but a ligand-dependent increase in GR phosphorylation may happen due to the dissociation of protein phosphatases [52]. The c-Jun N-terminal kinase (JNK) and extracellular signal-related kinase (ERK) regulate GR phosphorylation together, with JNK phosphorylating at site Ser246, which inhibits GR-mediated transcription activation, with ERK modulating GR phosphorylation indirectly through a GR-associated cofactor [54].

#### 5.2.3. Protein Phosphatases

In general, serine/threonine protein phosphatases (PP) influence GR phosphorylation negatively. Through phosphorylation of the Ser211 site, PP1α specifically has been found to enhance GR activity, suggesting that a disturbance of PP1α could lead to disrupted GR action and potentially contribute to disease [56]. PP5 behaves unlike PP1 and PP2A, and mainly acts in protein complexes, because its N-terminal folds over the catalytic site, which blocks access to substrates in the absence of proteins [53]. Changes in the concentrations of protein PP5 may differentially influence GR target gene expression.

PP5 negatively affects GR phosphorylation, and this may happen through PP5 dephosphorylating the Ser203, Ser211, and Ser226 sites, as reducing PP5 levels led to increased phosphorylation at those sites (Figure 2). Cytokines were also found to increase the presence of protein-phosphatase five (PP5), which modulates GR function, and PP5 suppression was shown to undo inhibition of GR phosphorylation at the Ser211 site [9]. Internally, the phosphorylation of the GR at certain serine residues is interdependent with phosphorylation at other serine residues, as GR phosphorylation at the Ser203 and Ser226 sites has been shown to be interdependent, such that one site serves as a gatekeeper for the other; Ser203 is more likely to be phosphorylated when Ser226 is not, and vice versa [52]. The GR ligand-binding domain (LBD) has been shown to regulate GR phosphorylation, as a GR derivative lacking the LBD demonstrated higher phosphorylation at Ser203, Ser211, and Ser226 sites [52].

### 5.3. Sex/Gender

In a stressed rat model, fluoxetine treatment affected GR levels and phosphorylation in hippocampal cells in a sex-specific manner. Specifically, the fluoxetine treatment showed opposite effects in the amount of phosphorylated GR (pGR) for pGR171 and pGR246 [57]. Rats exposed to chronic psychosocial isolation stress exhibited a disrupted nuclear pGR232-Cdk5 pathway and JNK signaling and responded to a concomitant fluoxetine treatment in a gender-specific manner. Fluoxetine treatment in females significantly decreased nuclear pGR232 levels, while in males, treatment did not significantly alter pGR232 levels. Both genders also differed in their pGR232/pGR246 ratio, with females exhibiting a predominance of the pGR246 isoform and males exhibiting a predominance of the pGR232 isoform. The effect of this difference in phosphorylated GR across genders may be a divergent regulation of GR-responsive gene regulation and differing cellular responses because of this [57,58]. In the case of breast cancer, estrogen has been shown to inhibit glucocorticoid activity through GR dephosphorylation via PP5 [53].

## 6. Modulatory Approaches and Drug Therapies Proposed to Tackle GR Phosphorylation

GR phosphorylation may be modulated through altering the activity of certain phosphatases targeting GR [52]. The protein phosphates 1, 2A, and 5, specifically, have been shown to have a role in GR function and phosphorylation, and inhibiting phosphatase activity through okadaic acid can result in hyperphosphorylation of GR, distributing GR from the nucleus to the cytoplasm, and inhibiting it from re-entering the nucleus [52]. GR phosphorylation by kinases such as CDKs, MAPKs, and GSK-3β modulates its function, and specific inhibitors such as JNK, ERK, and GSK-3β inhibitors could reduce GR phosphorylation activity at the sites Ser203, Ser226, and Ser404 [10]. GR activity could also be reduced by inhibitors of p38 MAPK or CDK, by reducing GR phosphorylation at sites like Ser211, which is required for full transcription [10].

### 6.1. pGR Agonist

The glucocorticoid agonists dexamethasone, prednisolone, and fluocinolone have been shown to promote GR phosphorylation at Ser211, and the antagonist RU486 very minimally stimulated phosphorylation at Ser211 and was able to effectively block dexamethasone-induced stimulation of Ser211 phosphorylation in a 10-fold excess [52], see Table 4. The glucocorticoid synthetic dexamethasone can cause immunosuppression, giving it the ability to inhibit immune and inflammatory responses [36]. Dexamethasone specifically regulates the key permeability factors angiopoietin-1 (Ang-1), angiopoietin-2 (Ang-2), and the vascular endothelial growth factor (VEGF) in three different cell types found in the BBB [22]. Dexamethasone was shown to upregulate Ang-1, which stabilizes the BBB, and overall downregulated VEGF, a permeabilizing actor [22]. Dexamethasone was also shown to increase effectiveness of BBB recovery in vitro after a primary blast injury by upregulating the zonula occludens (ZO)-1 tight junction protein, which strengthens and restores barrier properties through GR signaling [19], see Table 4. This may make GR regulation by dexamethasone a potential therapeutic target for disorders such as brain edema, autoimmune disorders such as rheumatoid arthritis, and diseases like MS which are characterized by a leaky BBB [19]. Dexamethasone was also shown to be an effective therapy for brain cancer, by reducing peritumoral edema and alleviating neurological symptoms [36]. However, dexamethasone and other corticosteroids can have numerous side effects when used in the long-term, such as immunosuppression, hyperglycemia, osteoporosis, and neuropsychiatric effects, instilling a need for balancing between therapeutic benefits and potential detriments, especially in patients going through long-term recovery [36].

### 6.2. pGR Antagonist and Modulators

Mifepristone, also known as RU486, is a potent glucocorticoid receptor (GR) antagonist that inhibits GR-mediated transactivation. The specificity of RU486’s mode of action in regulating GR phosphorylation of Ser211 or Ser206 still remains unclear. However, studies indicated that both early and late RU486 administration inhibited the elevated hippocampal FKBP4 level and hypothalamus GR level in a single prolonged stress rats in a posttraumatic stress disorder model [59], and early intervention with a GR antagonist aided in the correction of traumatic-stress-induced fear and anxiety dysregulation.

Dazucorilant (CORT113176) is a GR antagonist, selective, and high.affinity non-steroidal GR modulator which lacks cross-reactivity with other steroid receptors, making it more selective than RU486 (Table 4), and it has been shown to be effective in Alzheimer’s and amyotrophic lateral sclerosis models [44]. In a mouse model of Huntington’s Disease, CORT113176 was shown to lessen motor and neuropathological symptoms [44]. Treatment with CORT113176 was shown to reverse the increase in GR and MR, decreased the phosphorylation of GR, and normalized the HSP90/HSP70 ratio [60]. In addition, deregulation of the HPA axis and a feed-forward effect on prefrontal cortex GR sensitivity could participate in the etiology of AD, in perturbing Aβ and Tau homeostasis, with a report suggesting the reinforcement of the therapeutic potential of synaptic GR modulation in Alzheimer’s disease.

Antidepressant treatment may be an effective therapy regarding GR-HPA axis disorders, such as depression and stress-related disorders [1]. In a rat model exposed to chronic mild stress, antidepressant treatment with duloxetine was shown to normalize the effects caused by chronic mild stress, mainly in the prefrontal cortex by reducing the levels of the chaperone protein FKBP5, which is usually increased in expression for rats exposed to chronic mild stress, and normalizing alterations in FKBP5 mRNA and protein [44].

**Table 4:** Modulation of GR phosphorylation (pGR) and therapeutic applications.

Drug	Mechanism	Effects on GR Phosphorylation	Therapeutic Applications	References
Dexamethasone (Dex)	Full GR agonist binds to GR and activates anti-inflammatory and metabolic pathways	↑ pGR-Ser211 (↑ nuclear retention and transcription), ↓ pGR-Ser226 (↓ nuclear transport)	Brain edema, multiple sclerosis, brain tumors, increases BBB integrity (↑ ZO-1, ↑ occludin, ↑ claudin-5)	[19,22]
RU486 (Mifepristone)	GR antagonist prevents GR phosphorylation	RU486 has been shown to induce phosphorylation at Ser226 (transcriptional repression), but not Ser211 (transcriptional activation). With GR antagonist and CDK5 inhibitor, reduction in BDNF and cognitive dysfunction in aged mice were both rescued.	Inhibition of GR activation has potential for controlling postoperative cognitive dysfunction in aged individuals	[3,61]
CORT113176	Selective GR modulator targets GR without full activation	↓ pGR-Ser226 (restores GR function in stress-related disorders)	Reduces HPA axis hyperactivity, investigated for Huntington’s Disease, Amyotrophic Lateral Sclerosis, Alzheimer’s Disease	[44]

[Upregulation indicated by ↑ and downregulation indicated by ↓]. Abbreviations: Dex, Dexamethasone; pGR, GR phosphorylation: HPA axis, hypothalamic–pituitary–adrenal axis; DNA, deoxyribonucleic acid; ZO-1, Zonula Occludens-1; CDK5, Cyclin-dependent Kinase 5; BDNF, brain-derived neurotrophic factor.

## 7. Concluding Remarks and Future Perspective

In summary, GR phosphorylation is a complex process, playing an integral role in modulating glucocorticoids and influencing their therapeutic effectiveness in treating neurological disorders. The balance between phosphorylation and dephosphorylation activity among the GR’s specific serine residues modulates the GR’s transcriptional activity, nuclear localization, and interactions with co-factors. These factors shape the GR’s sensitivity and resistance in different neurological disorders. Current research poses multiple avenues to modulate GR phosphorylation, such as kinase inhibitors (JNK, ERK, p38 MAPK, GSK-3β inhibitors) for pharmacological therapies to restore GR function in the face of disorders like epilepsy, stroke, brain tumors, neurodegenerative disease, and psychiatric disorders. GR agonists like dexamethasone, and antagonists like RU486 or CORT113176, are also potential target therapies to overcome glucocorticoid resistance, stabilize the BBB, and modulate inflammatory and neurodegenerative processes. However, more research needs to be performed in the future to refine therapeutic interventions, and to understand how GR phosphorylation varies amongst tissues, cell types, and disease states, as GR phosphorylation is still in an emerging phase. Specific selective kinase and phosphatase modulators could be developed to fine-tune GR phosphorylation for specific therapeutic outcomes. Therapies could also take advantage of the benefits of glucocorticoids, while still considering possible detrimental steroid-related side effects. Overall, GR phosphorylation represents a promising and intricate therapeutic target for neurological disorders. Refining the understanding of GR phosphorylation and signaling pathways could bring future therapies which simultaneously optimize the therapeutic benefits of glucocorticoids, while minimizing adverse side effects, improving outcomes for patients with neurological disorders.

## Figures and Tables

**Table 1:** Summary of key GR phosphorylation sites and their effects on GR function.

GR Phosphorylation Sites	Function	References
Ser203	Inverse phosphorylation relationship with Ser226, cytoplasmic localization, may be prerequisite for Ser211 phosphorylation, may be required for full transcriptional activation	[3,10,15]
Ser211	Strongly linked to GR transcriptional activation, mainly nuclear localization, interdependent with Ser203 phosphorylation, may enhance interactions with transcriptional co-factors, phosphorylation required for full transcriptional activation	[3,10,15]
Ser226	Inverse phosphorylation relationship with Ser203, blocked phosphorylation may enhance GR’s transcriptional response, phosphorylation may inhibit GR function	[3,10,15]

Abbreviations: GR, glucocorticoid receptor

**Table 2:** Potential effects of GR phosphorylation on the neurovascular interface.

Process	Possible Effect on BBB	References
GR Activation (Not Directly Through Phosphorylation)	Enhances BBB integrity via upregulation of tight junction proteins (occludin, claudin-5, ZO-1)	[24]
GR Phosphorylation	Alters GR interaction with co-regulators and transcriptional activity, still needs further investigation	[14]
GR Phosphorylation in Neuroinflammation	Enhances or weakens neurovascular function, depending on GR phosphorylation state	[2,3,27]
GR Phosphorylation in Hypoxia	Promotes BBB protection or disruption, depending on dynamics of GR phosphorylation	[12,23]

Abbreviations: GR, glucocorticoid receptor; BBB, blood–brain barrier; ZO-1, zonula occludens-1.

## Abbreviations

The following abbreviations are used:

GR	Glucocorticoid Receptor
HPA	Hypothalamic–Pituitary–Adrenal
NTD	N-terminal domain
DNB	DNA binding domain
LBD	Ligand-binding domain
AF	Transactivation function
pGR	Phosphorylated GR
BBB	Blood–brain barrier
TLE	Temporal lobe epilepsy
LTP	Long term potentiation
HSP	Heat Shock Protein
Ang-1	Angiopoietin-1
VEGF	Vascular endothelial growth factor
BDNF	Brain-derived neurotrophic factor
ZO	Zonula Occludens
MS	Multiple Sclerosis
AD	Alzheimer’s Disease
GBM	Glioblastoma
HD	Huntington’s Disease
BD	Bipolar Disorder
Hap1	Huntingtin-associated protein 1
MDD	Major Depressive Disorder
ANXA1	Annexin A1
JNK	c-Jun N-terminal kinase
ERK	Extracellular signal-related kinase
CDK	Cyclin-dependent kinases
PP5	protein-phosphatase five
CNS	Central Nervous System
eNOS	endothelial nitric oxide synthase
MAPK	Mitogen-activated protein kinase
FKBP5	FK506 binding protein 51 also called FKBP5
